# Balancing Seizure Quality and Cardiovascular Safety in Anesthetic Selection During ECT

**DOI:** 10.1192/j.eurpsy.2026.10438

**Published:** 2026-07-31

**Authors:** V. Patel, J. D. White, J. D. Hagan, B. R. Carr

**Affiliations:** 1 KPCOM, Fort Lauderdale; 2 University of Florida, Gainesville, United States

## Abstract

**Introduction:**

Electroconvulsive therapy (ECT) is a cornerstone of treatment for severe depression, suicidality, and catatonia. Anesthetic choice directly influences seizure threshold, seizure quality, hemodynamic stability, and ultimately therapeutic efficacy. Cardiovascular comorbidity amplifies these challenges. For psychiatrists, acquiring fluency in anesthetic principles contributes to safety, tolerability, and quality outcomes.

**Objectives:**

To review anesthetic agents used in ECT and clarify how pharmacological selection intersects with treatment efficacy and patient safety.

**Methods:**

Narrative educational review of psychiatric, anesthetic, pharmacologic literature (2010–2025), focus on four mainstay agents showcasing the effect on hemodynamics, cardiac function, and therapeutic seizure characteristics.

**Results:**

ECT anesthetics differ substantially in both neuropsychiatric and cardiovascular profiles (Table 1):

Methohexital is the gold standard: it lowers seizure threshold, sustains adequate seizure duration, and provides remarkable hemodynamic stability in the majority of patients. However, its dose-dependent negative inotropy can become significant in patients with advanced heart failure.

Etomidate preserves cardiac output and reliably produces long seizure durations, often yielding higher efficacy in treatment-resistant depression. Downsides include temporary adrenal suppression, especially in the context of frequent treatments and limited availability outside North America.

Propofol blunts hypertensive surges and provides rapid emergence, though slower than methohexital. It raises seizure threshold and shortens seizure duration. Hemodynamic effects include vasodilation leading to lower systemic vascular resistance and some negative inotropy, making it less suitable in patients with heart failure.

Ketamine prolongs seizure duration and augments sympathetic tone, occasionally improving mood acutely. Yet its indirect sympathetic activation may be destabilizing in patients with arrhythmias or advanced coronary disease.

**Table 1.** Table 1. Long description.

	Onset time (s) (IV)	Seizure Threshold	Context-sensitive half time 1 hour infusion (min)	Half-life (hours)	Effect on LVEF
Methohexital	~30	← → “Gold Standard”	Less than propofol	2-6	↓↓
Propofol	40-60	↑	10	4-7	↓
Ketamine	10-30	↑	~10	2.5-2.8	←→/↑
Etomidate	40-60	↓	8 (bolus dose)	2.9-5.3	←→/↑

**Conclusions:**

Psychiatrists who understand anesthetic pharmacology can better anticipate treatment trajectories and maintain treatment efficacy and safety, especially in patients with cardiovascular comorbidities. Education in anesthetic-psychiatric intersections within the psychiatric curriculum would benefit safe ECT therapy in cardiac patients.

**Disclosure of Interest:**

None Declared

## Long descriptions

### Table 1. Long description

The table consists of six columns and five rows.

Column headers from left to right are: Drug Name, Onset time in seconds I V, Seizure Threshold, Context-sensitive half time 1 hour infusion in minutes, Half-life in hours, and Effect on L V E F.

Row 1: Methohexital. Onset time is approximately 30. Seizure Threshold is indicated by a left-right arrow and the text Gold Standard. Context-sensitive half time is less than propofol. Half-life is 2 to 6. Effect on L V E F is indicated by two downward arrows.

Row 2: Propofol. Onset time is 40 to 60. Seizure Threshold is indicated by an upward arrow. Context-sensitive half time is 10. Half-life is 4 to 7. Effect on L V E F is indicated by a single downward arrow.

Row 3: Ketamine. Onset time is 10 to 30. Seizure Threshold is indicated by an upward arrow. Context-sensitive half time is approximately 10. Half-life is 2.5 to 2.8. Effect on L V E F is indicated by a left-right arrow forward slash upward arrow.

Row 4: Etomidate. Onset time is 40 to 60. Seizure Threshold is indicated by a downward arrow. Context-sensitive half time is 8 for a bolus dose. Half-life is 2.9 to 5.3. Effect on L V E F is indicated by a left-right arrow forward slash upward arrow.

Navigate back to Table 1..

